# Use of Smartphones to Detect Diabetic Retinopathy: Scoping Review and Meta-Analysis of Diagnostic Test Accuracy Studies

**DOI:** 10.2196/16658

**Published:** 2020-05-15

**Authors:** Choon Han Tan, Bhone Myint Kyaw, Helen Smith, Colin S Tan, Lorainne Tudor Car

**Affiliations:** 1 Lee Kong Chian School of Medicine Nanyang Technological University Singapore Singapore; 2 Centre for Population Health Sciences, Lee Kong Chian School of Medicine, Nanyang Technological University Singapore Singapore; 3 Family Medicine and Primary Care, Lee Kong Chian School of Medicine, Nanyang Technological University Singapore Singapore; 4 Department of Ophthalmology Tan Tock Seng Hospital Singapore Singapore; 5 Department of Primary Care and Public Health School of Public Health Imperial College London London United Kingdom

**Keywords:** diabetic retinopathy, smartphone, mobile phone, ophthalmoscopy, artificial intelligence, telemedicine

## Abstract

**Background:**

Diabetic retinopathy (DR), a common complication of diabetes mellitus, is the leading cause of impaired vision in adults worldwide. Smartphone ophthalmoscopy involves using a smartphone camera for digital retinal imaging. Utilizing smartphones to detect DR is potentially more affordable, accessible, and easier to use than conventional methods.

**Objective:**

This study aimed to determine the diagnostic accuracy of various smartphone ophthalmoscopy approaches for detecting DR in diabetic patients.

**Methods:**

We performed an electronic search on the Medical Literature Analysis and Retrieval System Online (MEDLINE), EMBASE, and Cochrane Library for literature published from January 2000 to November 2018. We included studies involving diabetic patients, which compared the diagnostic accuracy of smartphone ophthalmoscopy for detecting DR to an accurate or commonly employed reference standard, such as indirect ophthalmoscopy, slit-lamp biomicroscopy, and tabletop fundus photography. Two reviewers independently screened studies against the inclusion criteria, extracted data, and assessed the quality of included studies using the Quality Assessment of Diagnostic Accuracy Studies–2 tool, with disagreements resolved via consensus. Sensitivity and specificity were pooled using the random effects model. A summary receiver operating characteristic (SROC) curve was constructed. This review is reported in line with the Preferred Reporting Items for a Systematic Review and Meta-analysis of Diagnostic Test Accuracy Studies guidelines.

**Results:**

In all, nine studies involving 1430 participants were included. Most studies were of high quality, except one study with limited applicability because of its reference standard. The pooled sensitivity and specificity for detecting any DR was 87% (95% CI 74%-94%) and 94% (95% CI 81%-98%); mild nonproliferative DR (NPDR) was 39% (95% CI 10%-79%) and 95% (95% CI 91%-98%); moderate NPDR was 71% (95% CI 57%-81%) and 95% (95% CI 88%-98%); severe NPDR was 80% (95% CI 49%-94%) and 97% (95% CI 88%-99%); proliferative DR (PDR) was 92% (95% CI 79%-97%) and 99% (95% CI 96%-99%); diabetic macular edema was 79% (95% CI 63%-89%) and 93% (95% CI 82%-97%); and referral-warranted DR was 91% (95% CI 86%-94%) and 89% (95% CI 56%-98%). The area under SROC curve ranged from 0.879 to 0.979. The diagnostic odds ratio ranged from 11.3 to 1225.

**Conclusions:**

We found heterogeneous evidence showing that smartphone ophthalmoscopy performs well in detecting DR. The diagnostic accuracy for PDR was highest. Future studies should standardize reference criteria and classification criteria and evaluate other available forms of smartphone ophthalmoscopy in primary care settings.

## Introduction

Diabetic retinopathy (DR) is the leading cause of impaired vision worldwide [1]. One in three patients with diabetes mellitus (DM) have DR [2]. DR includes proliferative DR (PDR) and various levels of nonproliferative DR (NPDR). PDR, characterized by retinal neovascularization at the disc and elsewhere, displays signs of angiogenesis in response to retinal tissue hypoxia. Neovascularization potentially leads to preretinal and vitreous hemorrhage, resulting in visual loss and, eventually, tractional retinal detachment. It may also cause iris neovascularization with resultant increase in intraocular pressure, eventually leading to neovascular glaucoma [3]. Typical clinical features of NPDR include the following: (1) microaneurysms and intraretinal hemorrhages from weak capillary walls; (2) hard exudates from vascular protein leakage; and (3) cotton wool spots, caused by ischemic infarcts leading to fluid accumulation. Diabetic macular edema (DME), caused by the thickening of and fluid accumulation in the retina, can occur at any stage of DR [4].

Diabetic eye disease is treatable. Treatments include vascular endothelial growth factor inhibitors, panretinal or focal photocoagulation, and vitrectomy [5]. Strict glycemic and blood pressure control can also delay the development of DR or reduce DR severity [6]. Treatments available are more effective at halting or slowing visual loss than reversing visual impairment. Yet, most patients remain asymptomatic until the advanced stages of DR. Therefore, early detection of DR before irreversible loss of visual acuity is crucial to ensure better patient outcomes [7].

The gold standard diagnostic test for DR is the Early Treatment Diabetic Retinopathy Study (ETDRS) 7-field stereoscopic color fundus photography or fluorescein angiography [8]. However, fundus cameras are nonportable, expensive, and operator dependent, often requiring patients to sit upright [9,10]. Moreover, fluorescein angiography is invasive, costly, and associated with prominent side effects [11]. Thus, they are impractical for screening in primary care or mobile settings. Other accurate [12] and frequently employed DR identification approaches include the following: (1) ophthalmoscopy; (2) slit-lamp biomicroscopy; and (3) other forms of fundus photography [13]. Optical coherence tomography is an emerging technology that reliably identifies DME by quantifying retinal thickness [14], but it is expensive and bulky and it cannot accurately grade DR severity.

Smartphone ophthalmoscopy, the use of a smartphone’s in-built camera for retinal imaging, could be a valuable method for detecting DR because of its affordability, portability, and ease of use compared with traditional approaches [15]. Various health care workers could potentially operate a smartphone-based retinal imaging device, without limiting this procedure to highly specialized staff. Images acquired by smartphones can be easily shared with and graded remotely by ophthalmologists or other trained graders via telemedicine. These benefits are particularly important in resource-constrained health care settings, such as rural areas in developing countries lacking medical equipment and trained health care professionals [16]. Several literature reviews [17-19] have discussed smartphone retinal imaging technology and underscored the huge potential of smartphone ophthalmoscopy for detecting DR. Given the potential of this novel approach, we performed a scoping review to systematically collate and assess evidence regarding the accuracy of smartphone ophthalmoscopy for DR identification.

## Methods

### Reporting Guidelines

This scoping review was reported in line with the Preferred Reporting Items for a Systematic Review and Meta-analysis of Diagnostic Test Accuracy Studies (PRISMA-DTA) guidelines [20] and conducted according to the Cochrane Handbook for Systematic Reviews of Diagnostic Test Accuracy [21]. We adopted a scoping review approach [22,23] because of a broad set of inclusion criteria. The protocol for this review was published in *BMJ Open* [24]. We were unable to register this protocol with PROSPERO as it does not include scoping reviews.

### Search Strategy

We performed a librarian-assisted search on the Medical Literature Analysis and Retrieval System Online (MEDLINE) (Ovid), EMBASE (Ovid), and the Cochrane Library for papers published from January 2000 to November 2018. Articles published before 2000 were excluded because before that smartphone technology was limited. We used both medical subject headings (MeSH) and keywords relating to DR (eg, “diabetic retinopathy,” “macular edema,” and “diabetic maculopathy”) and to smartphones (eg, “mobile health,” “mobile phones,” and “applications”) or AI (eg, “artificial intelligence” and “machine learning”; Multimedia Appendix 1). We also explored the bibliography of both primary articles and reviews to identify potentially eligible studies missed by the electronic search.

### Study Selection

The inclusion criteria were as follows: (1) studies evaluating the diagnostic test accuracy of smartphone ophthalmoscopy for detecting DR in patients with type 1 or 2 DM; (2) studies utilizing a smartphone’s in-built camera for retinal imaging, including the use of any attachments externally fitted to the smartphone; (3) studies comparing smartphone ophthalmoscopy with any acceptable and commonly employed reference standard, such as fundus photography, indirect ophthalmoscopy, slit-lamp biomicroscopy, or fluorescein angiography; (4) studies employing any kind of health care professional to acquire the smartphone images. Language was not an exclusion criterion.

Examples of eligible smartphone ophthalmoscopy techniques include the following:

- Direct ophthalmoscopy: An adaptor is externally attached to a smartphone’s camera. These adaptors usually contain polarizers that reduce artifacts from corneal reflections. The arrangement of polarizers, beam-splitters, and lenses produces an annular illumination pattern.
- Indirect ophthalmoscopy: This simpler, monocular design involves a single lens (eg, 20 D condenser) placed between the smartphone camera and eye. It can be mounted on the phone via hardware or manually held in position.

Covidence software (Veritas Health Innovation, Melbourne, Australia) was used to remove duplicated studies [25]. After a pilot screening of 20 citations to calibrate the judging criteria, two reviewers independently screened all articles retrieved from the search strategy by title and abstract, using Covidence. Subsequently, we screened the full text of the remaining articles and performed data extraction using a prepiloted form. Any disagreements were resolved through consensus.

### Data Collection

A data extraction form (Multimedia Appendix 2) was created and piloted to record the following data from each study: (1) study author and date published; (2) sample size; (3) participant characteristics (eg, age, duration and type of DM); (4) information regarding imaging techniques (eg, details about smartphones and adaptors used, image resolution); (5) health care professional performing smartphone ophthalmoscopy; (6) reference standard used; and (7) test results (eg, true positives [TP], false positives [FP], true negatives [TN], and false negatives [FN]). Corresponding authors were contacted for additional details or missing data required to construct a 2×2 table. Two reviewers independently extracted study data using a data extraction template created in Microsoft Excel, with disagreements resolved via consensus.

### Quality Assessment

The Quality Assessment of Diagnostic Accuracy Studies tool, QUADAS-2, consisting of descriptions and signaling questions, was used to assess the risk of bias and applicability of all included studies in four domains pertaining to (1) patient selection, (2) index test, (3) reference standard, and (4) flow of participants through the study and timing between the index test and reference standard [26]. Two reviewers independently assessed study quality, and disagreements were resolved via discussion until a consensus was reached.

### Statistical Analysis

We constructed 2×2 tables based on data from each study. The sensitivity, specificity, positive likelihood ratio (LR+), negative likelihood ratio (LR−), diagnostic odds ratio (DOR), and area under summary receiver operating characteristic (SROC) curve were calculated using a random effects model because of the high expected heterogeneity [27]. We constructed SROC using the bivariate model where possible. Being both a hierarchical and random effects model, the bivariate model is preferred to the Moses-Littenberg SROC curve—the former method accounts for between-study heterogeneity. For SROC curves employing the bivariate model, elliptical 95% confidence regions were obtained by joining the individual confidence regions for logit sensitivity and logit specificity via parametric representations [28,29].

Heterogeneity was evaluated using chi-square (χ²) and I^2^ values of likelihood ratio tests (LRT) or DOR, with I^2^<25%, 25–75%, and >75% representing low, moderate, and high degree of inconsistency, respectively. Threshold effect was measured using the Spearman correlation coefficient ρ between logits of sensitivity and specificity, with ρ closer to −1 indicating higher threshold effect and better fit of the SROC curve. If information regarding a condition’s prevalence was available from the literature, we calculated the posttest probability using the Fagan nomogram. A *P*<.05 was considered statistically significant. All analyses were performed using Review Manager version 5.3 from the Cochrane Collaboration [30], METANDI and MIDAS commands in Stata 15.1 (StataCorp, College Station, Texas), Meta-Disc version 1.4 (Ramón y Cajal Hospital, Madrid, Spain) [31], and *mada* package in R.

## Results

### Study Selection and Study Characteristics

**Figure 1:**
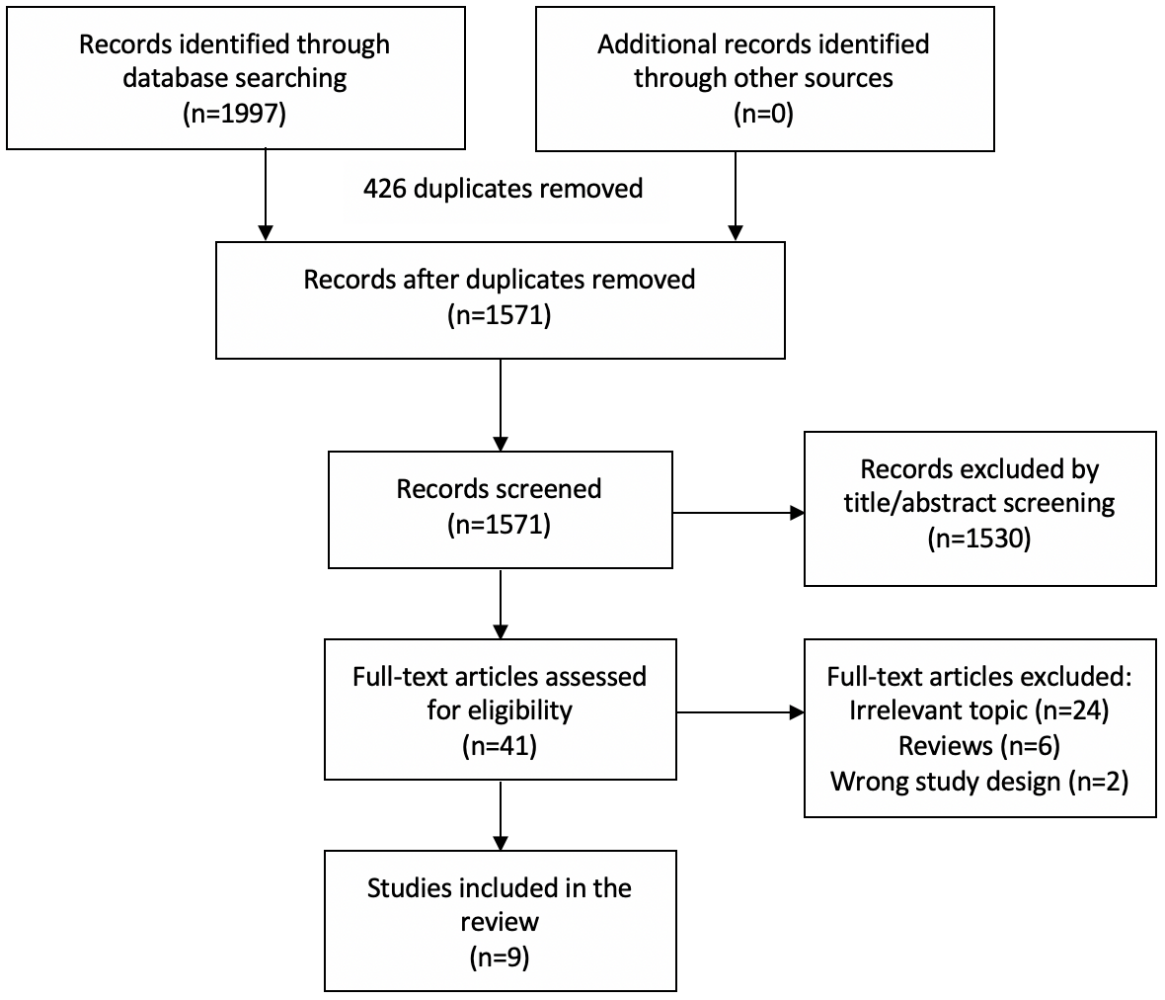
Flowchart depicting the identification of relevant studies.

Our search strategy yielded 1571 unique records. Of those records, the full text for 41 articles was assessed, and nine studies [32-40] met the inclusion criteria (Figure 1). Two [32,33] of the included studies were conference abstracts. A total of 1430 diabetic patients (at least 2743 eyes) were recruited among these studies.

All studies reported smartphone fundoscopy techniques involving mydriatic, color, and nonstereoscopic imaging (Tables 1 and 2, Multimedia Appendix 3). A total of four studies originated from India, three studies from the United States, and one from Italy. All studies which reported data on gender recruited both males and females. These fundus photographs were graded by ophthalmologists, retinal specialists, or artificial intelligence (AI). In all, seven studies utilized direct ophthalmoscopy to acquire smartphone fundus images, while two studies [38,40] used indirect ophthalmoscopy.

A total of five studies [32,33,37,39,40] employed slit-lamp biomicroscopy as the reference standard, of which two studies complemented the examination with dilated indirect ophthalmoscopy [39,40]. In all, two studies [35,38] utilized 7-field mydriatic fundus photography using a tabletop fundus camera; one study [34] used *traditional in-clinic diagnosis* including a dilated eye examination; and one study [36] utilized ophthalmologists’ grading of the same smartphone-acquired images as the reference standard. Overall, four studies [32,36,37,40] utilized the International Clinical DR Disease Severity Scale to grade DR; three studies [34,35,38] employed the Airlie House or modified ETDRS criteria; and one study [39] used the United Kingdom’s National Health Service (NHS) guidelines. Referral-warranted DR (RWDR) was defined as moderate NPDR or worse or DME; vision-threatening DR (VTDR) as severe NPDR, PDR, or DME; and sight-threatening DR (STDR) as PDR or DME. Health professionals performing smartphone ophthalmoscopy included medical students, interns or assistants, retinal specialists, ophthalmologists, and ophthalmic photographers. Most studies reported no funding sources or conflicting interests, while such information was unavailable for two studies [32,33]. In all, two studies [34,40] received funding, one of which disclosed multiple authors holding positions in DigiSight Technologies, Inc.

**Table 1:** Characteristics of included studies.

Study author, year	Country, setting	Sample size (patients/eyes)	Age (years), mean (SD)	Diabetes duration (years)		Diabetic retinopathy severity scale		Reference standard	
				Mean (SD)	Range				
Bhat, 2016 [32]	N/A^a^	80/N/A	N/A	N/A	N/A		ICDR^b^ severity scale; no referral defined as no or mild signs of DR^c^.		Slit-lamp exam
Kim, 2017 [33]	United States, Retina Clinic	72/144	N/A	N/A	N/A		Referable DR defined as moderate NPDR^d^ or worse, or DME^e^.		Slit-lamp biomicroscopy
Kim, 2018 [34]	United States, Retina Clinic	71/142	56.7 (16.9)	N/A	N/A		Airlie House ETDRS^f^ criteria; RWDR^g^ defined as moderate NPDR or worse, or DME.		Gold standard dilated eye examination, with optical coherence tomography for DME
Rajalakshmi, 2015 [35]	India, Tertiary care diabetes hospital	301/602	53.5 (9.6)	12.5 (7.3)	N/A		Modified ETDRS criteria; STDR^h^ defined as PDR^i^ or DME		Mydriatic 7-standard field digital retinal photography
Rajalakshmi, 2018 [36]	India, Tertiary care diabetes hospital	301/602	N/A	N/A	N/A		ICDR severity scale; STDR defined as severe NPDR, PDR, or DME; RDR^j^ defined as moderate NPDR or worse, or DME.		Remidio Fundus On Phone images graded by ophthalmologists
Russo, 2015 [37]	Italy, Diabetic center	120/240	58.8 (16.4)	11.6 (9.7)	N/A		ICDR severity scale; ETDRS criteria for DME; RWDR defined as moderate NPDR or worse, regardless of DME status.		Dilated slit-lamp biomicroscopy by a retinal specialist
Ryan, 2015 [38]	India, Ophthalmology clinic of a tertiary diabetes care center	300/600	48.0 (11.0)	N/A	0.1-37.2 years		Modified ETDRS criteria; VTDR^k^ defined as severe NPDR or worse, or DME.		Mydriatic 7-field fundus photography by trained optometrists
Sengupta, 2018 [39]	India, Aravind Eye Hospital	135/233	54.1 (8.3)	10.7 (5.1)	N/A		National Health Service guidelines; VTDR defined as R2-level or worse (severe NPDR, PDR), or DME.		Dilated slit-lamp biomicroscopy (+90 D lens) and indirect ophthalmoscopy by retinal specialists
Toy, 2016 [40]	United States, Health care safety-net ophthalmology clinic	50/100	60.5 (10.6)	11.9 (8.4)	N/A		ICDR severity scale; RWDR defined as moderate NPDR or worse, or ungradable images.		Slit-lamp exam + dilated ophthalmoscopy by technicians

^a^N/A: not available.

^b^ICDR: International Clinical Diabetic Retinopathy.

^c^DR: diabetic retinopathy.

^d^NPDR: nonproliferative diabetic retinopathy.

^e^DME: diabetic macular edema.

^f^ETDRS: Early Treatment Diabetic Retinopathy Study.

^g^RWDR: referral-warranted diabetic retinopathy.

^h^STDR: sight-threatening diabetic retinopathy.

^i^PDR: proliferative diabetic retinopathy.

^j^RDR: referable diabetic retinopathy.

^k^VTDR: vision-threatening diabetic retinopathy.

**Table 2:** Description of smartphone ophthalmoscopy imaging techniques.

Study author, year	Attachment used	Imaging technique	Smartphone used	Ungradable
Bhat, 2016 [32]	Ocular Cellscope	Up to five fields, 50°; AI^a^**:** EyeArt v1.2 software used to grade images; acquired by: medical interns and assistants.	iPhone 5S	N/A^b^
Kim, 2017 [33]	Cellscope Retina	Both human and AI (EyeApp) graders employed.	N/A	N/A
Kim, 2018 [34]	Cellscope Retina	5-field, 50°; fields imaged: central, inferior, superior, nasal, and temporal retina; images were digitally stitched, creating a 100° image; pixels per retinal degree: 52.3; acquired by: medical students or interns.	iPhone 5S	2 (1.7%) images/eyes
Rajalakshmi, 2015 [35]	Remidio Fundus on Phone (FOP)	4-field, 45°; fields imaged: macula, disc and nasal to optic disc, superior-temporal, inferior-temporal retina; autofocus function of smartphone was used.	Android phone	0
Rajalakshmi, 2018 [36]	Remidio Fundus on Phone (FOP)	4-field, 45°; fields imaged: macula centered, disc centered, superior-temporal, and inferior-temporal retina; AI: EyeArt software used to grade images.	Android phone	5 (1.7%) patients
Russo, 2015 [37]	D-Eye (Si14 SpA, Padova, Italy)	20°; videography and digital images acquired, comprising the posterior pole, macula, optic disc, and peripheral retina; resolution: 3264×2448 pixels; pixels per retinal degree: 150; acquired by: a retinal specialist.	iPhone 5	9 (3.8%) eyes
Ryan, 2015 [38]	20 D lens	Videography and then screenshots to obtain the best images of optic nerve and macula; resolution: 3264×2488 pixels; FilmIc Pro app used to adjust focus and zoom independently; acquired by: a medical student with limited training.	iPhone 5	11 (1.8%) photographs
Sengupta, 2018 [39]	Remidio FOP	3-field, 45°; fields imaged: posterior pole (macula centered), nasal, and superotemporal field; resolution: 441 pixels per inch; acquired by: ophthalmic photographer without special training.	HTC One (M8)	1.7-2.1% of images
Toy, 2016 [40]	Volk Digital ClearField lens mounted on Paxos Scope posterior segment hardware adapter	Variable number of fields, 45°; acquired by: an ophthalmologist.	iPhone 5S	2 (2%) eyes

^a^AI: artificial intelligence.

^b^N/A: not available.

### Quality Assessment

**Figure 2:**
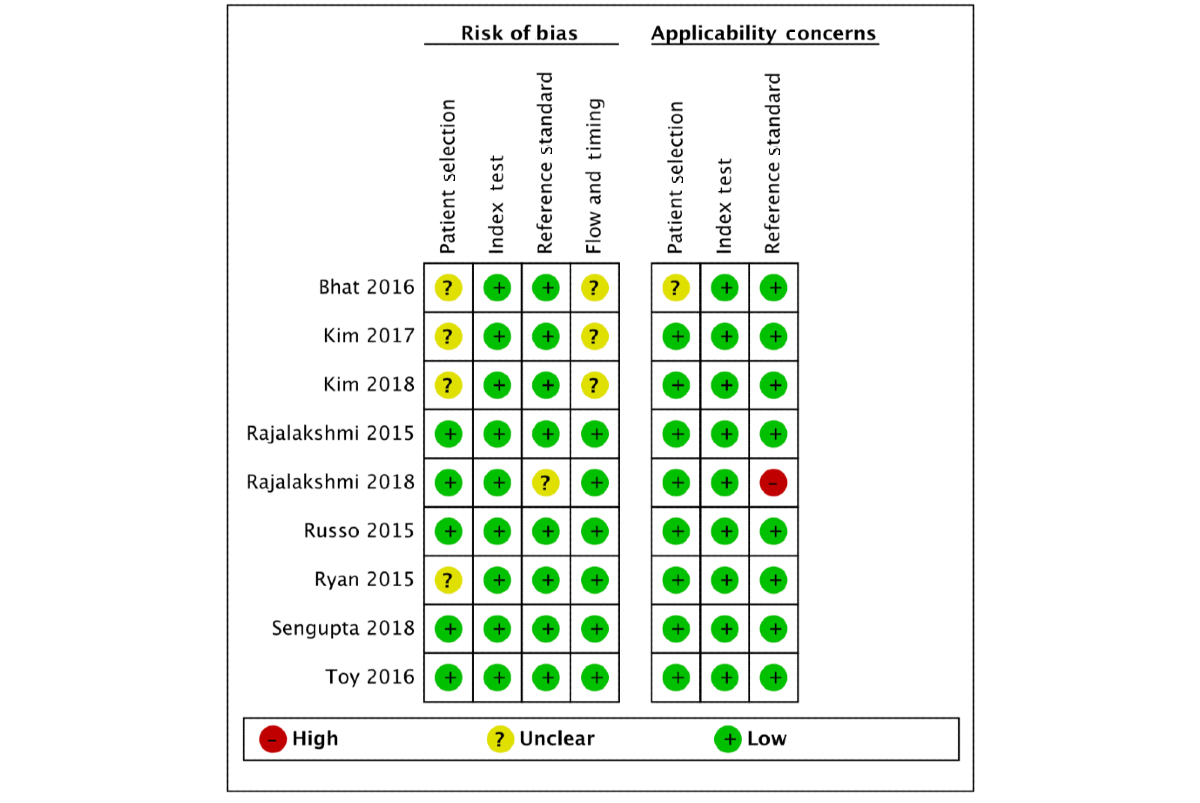
Quality of included studies assessed via Quality Assessment of Diagnostic Accuracy Studies–2 tool.

We carried out the quality assessment of the included studies using the QUADAS-2 criteria (Figure 2, Multimedia Appendix 4). Most studies were of high quality with low risk of bias and applicability concerns. A total of four studies had an unclear risk of bias for patient selection because of the lack of information regarding patient sampling or inappropriate exclusions. The two abstracts were of lower quality than the other studies because of the limited amount of information available. One study contained applicability concerns because it employed ophthalmologists’ grading of smartphone fundoscopy images as the reference standard; it was excluded from the meta-analysis.

### Meta-Analysis

#### Any Diabetic Retinopathy

**Figure 3:**
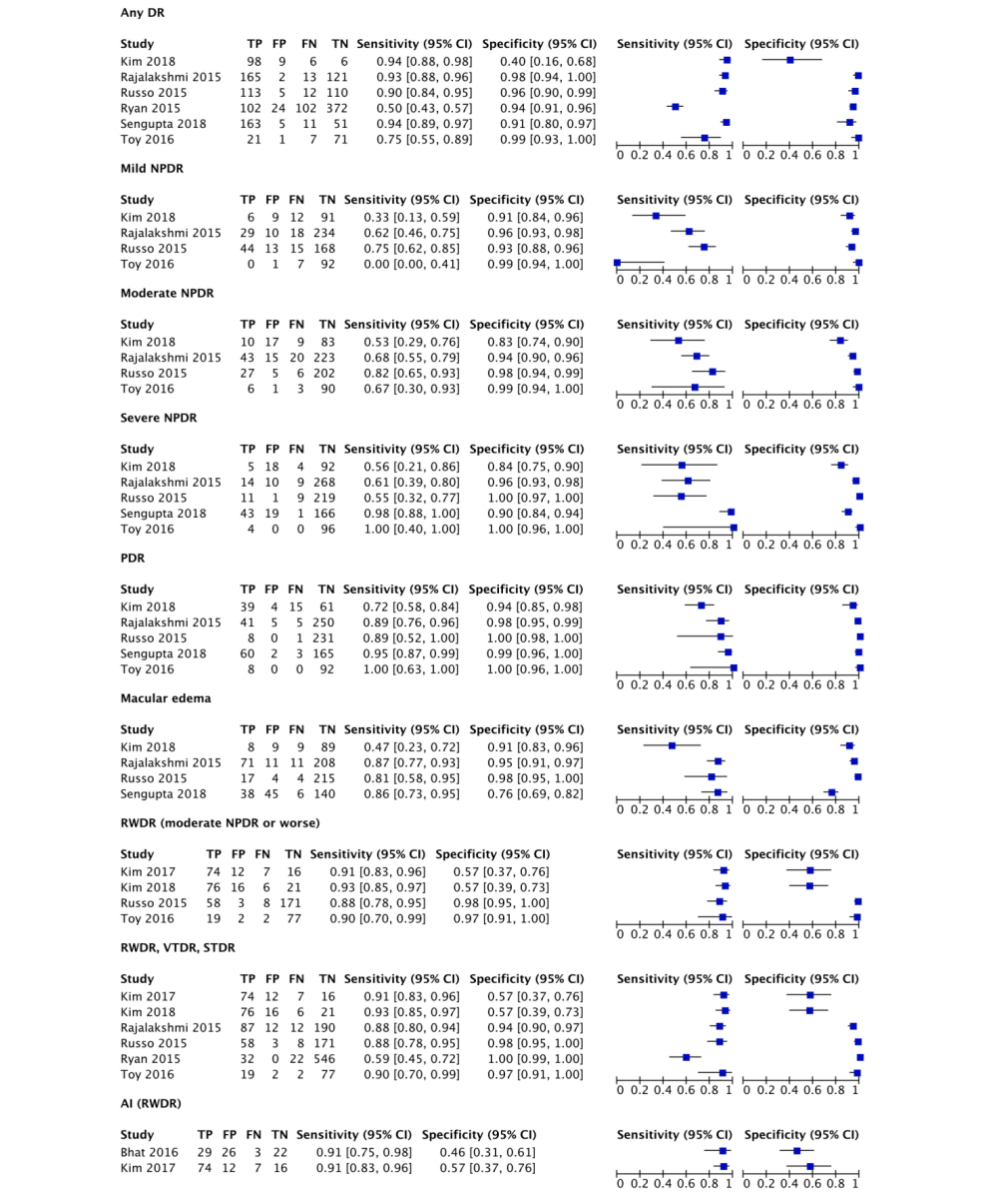
Forest plot of the sensitivity and specificity of smartphone ophthalmoscopy in detecting different grades of diabetic retinopathy. AI: artificial intelligence; FN: false negatives; FP: false positives; NPDR: nonproliferative diabetic retinopathy; PDR: proliferative diabetic retinopathy; RWDR: referral-warranted diabetic retinopathy; STDR: sight-threatening diabetic retinopathy; TN: true negatives; TP: true positives; VTDR: vision-threatening diabetic retinopathy.

**Figure 4:**
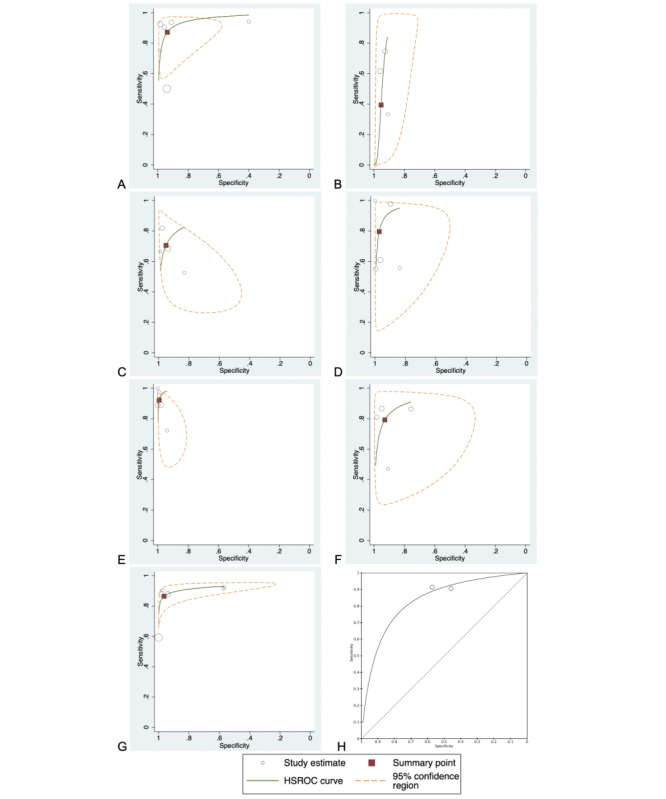
Summary receiver operating characteristic curves of smartphone ophthalmoscopy in detecting (A) any diabetic retinopathy; (B) mild nonproliferative diabetic retinopathy; (C) moderate nonproliferative diabetic retinopathy; (D) severe nonproliferative diabetic retinopathy; (E) proliferative diabetic retinopathy; (F) diabetic macular edema; (G) referral-warranted diabetic retinopathy, vision-threatening diabetic retinopathy, or sight-threatening diabetic retinopathy; (H) artificial intelligence to detect referral-warranted diabetic retinopathy. HSROC: hierarchical summary receiver operating characteristic.

In all, six studies (977 participants; Figures 3 and 4; Table 3) presented data on detecting any DR [34,35,37-40]. I^2^_LRT_ was 96.8% (95% CI 94.6%-99.1%), χ²_5_=63.3, and ρ=−0.332. DOR was 100 (95% CI 27.4-368). Sensitivity and specificity ranged from 50% to 94% and 40% to 99%, respectively. The pooled sensitivity was 87.1% (95% CI 73.9%-94.2%); pooled specificity was 93.7% (95% CI 80.9%-98.1%). LR+ was 13.8 (95% CI 4.37-43.6); LR− was 0.138 (95% CI 0.066-0.287). The area under curve (AUC) was 0.957 (95% CI 0.936-0.972). Considering a pretest probability of 35.4% in diabetic patients [2], using the Fagan nomogram, the posttest probability for a positive and negative result was 88% and 7%, respectively.

We performed subgroup analysis by removing studies individually and investigating the effect on both I^2^ and ρ. When one study [38] was removed, I^2^ decreased to 93.0% (95% CI 86.8%-99.3%) and ρ decreased to −1.00, implying this study contributed to the heterogeneity. However, both the type of ophthalmoscopy (direct vs indirect) and reference standard used did not account for the heterogeneity.

**Table 3:** Summary of smartphone ophthalmoscopy’s test accuracy in detecting different grades of diabetic retinopathy.

DR^a^ staging	Studies, n	Overall pooled sensitivity, % (95% CI)	Overall pooled specificity, % (95% CI)	Positive likelihood ratio (95% CI)	Negative likelihood ratio (95% CI)	Diagnostic odds ratio (95% CI)	Area under summary receiver operating characteristic curve (95% CI)
Any DR	6	87 (74-94)	94 (81-98)	14 (4.4–44)	0.14 (0.06-0.29)	100 (27.4-368)	0.957 (0.936-0.972)
Mild NPDR^b^	4	39 (10-79)	95 (91-98)	8.6 (3.6-20)	0.64 (0.32-1.3)	13.6 (3.14-58.5)	0.939 (0.915-0.957)
Moderate NPDR	4	71 (57-81)	95 (88-98)	15 (4.9-43)	0.31 (0.20-0.49)	46.9 (10.6-208)	0.879 (N/A)
Severe NPDR	5	80 (49-94)	97 (88-99)	28 (6.1-133)	0.21 (0.069-0.65)	134 (17.5-1040)	0.965 (0.945-0.978)
PDR^c^	5	92 (79-97)	99 (96-99)	97 (22-425)	0.079 (0.027-0.23)	1225 (117-12,800)	0.979 (N/A)
DME^d^	4	79 (63-89)	93 (82-97)	11 (4.2-30)	0.22 (0.12-0.42)	49.8 (13.7-180)	0.925 (0.898-0.945)
RWDR^e^ (moderate NPDR or worse)	4	91 (86-94)	89 (56-98)	8.1 (1.6-41)	0.11 (0.072-0.16)	75.8 (13.9-414)	0.921 (0.894-0.941)
RWDR, VTDR^f^, STDR^g^	6	87 (77-92)	96 (71-99)	24 (2.6-226)	0.14 (0.087-0.23)	171 (25.9-1142)	0.929 (0.903-0.949)
AI^h^ (RWDR)	2	91 (84-96)	50 (38-62)	1.8 (1.4-2.3)	0.17 (0.088-0.32)	11.3 (4.92-26.1)	N/A^i^

^a^DR: diabetic retinopathy.

^b^NPDR: nonproliferative diabetic retinopathy.

^c^PDR: proliferative diabetic retinopathy.

^d^DME: diabetic macular edema.

^e^RWDR: referral-warranted diabetic retinopathy.

^f^VTDR: vision-threatening diabetic retinopathy.

^g^STDR: sight-threatening diabetic retinopathy.

^h^AI: artificial intelligence.

^i^N/A: not available.

#### Mild Nonproliferative Diabetic Retinopathy

In all, four studies (542 participants) presented data on detecting mild NPDR [34,35,37,40]. I^2^_LRT_ was 81.5% (95% CI 60.6%-100%), χ²_3_=10.8, and ρ=−0.862. DOR was 13.6 (95% CI 3.14-58.5). Sensitivity and specificity ranged from 0% to 75% and 91% to 99%, respectively. The pooled sensitivity was 39.4% (95% CI 10.1%-79.0%); pooled specificity was 95.4% (95% CI 91.3%-97.6%). LR+ was 8.60 (95% CI 3.64-20.3); LR− was 0.635 (95% CI 0.323-1.25). One study [40] using a lens mounted on Paxos scope yielded a sensitivity of 0%. The AUC was 0.939 (95% CI 0.915-0.957).

#### Moderate Nonproliferative Diabetic Retinopathy

A total of four studies (542 participants) presented data on detecting moderate NPDR [34,35,37,40]. DOR was 46.9 (95% CI 10.6-208; I^2^_DOR_=85.4%; χ²_3_=20.6). Sensitivity and specificity ranged from 53% to 82% and 83% to 99%, respectively. The pooled sensitivity was 70.5% (95% CI 56.6%-81.4%); pooled specificity was 95.1% (95% CI 87.8%-98.2%). LR+ was 14.5 (95% CI 4.89-43.2); LR− was 0.310 (95% CI 0.195-0.492). The AUC was approximately 0.879.

One study [39] assessed the sensitivity and specificity of smartphone ophthalmology in detecting R1 disease (ie, mild and moderate NPDR) to be 88.2% (95% CI 85.7%-91.6%) and 83.4% (95% CI 78%-87%), respectively.

#### Severe Nonproliferative Diabetic Retinopathy

Overall, five studies (677 participants) presented data on detecting severe NPDR [34,35,37,39,40]. I^2^_LRT_ was 94.0% (95% CI 88.9%-99.1%), χ²_4_=33.4, and ρ=−0.111. DOR was 134 (95% CI 17.5-1039). Sensitivity and specificity ranged from 55% to 100% and 84% to 100%, respectively. The pooled sensitivity was 79.5% (95% CI 48.6%-94.1%); pooled specificity was 97.1% (95% CI 87.7%-99.4%). LR+ was 28.4 (95% CI 6.06-133); LR− was 0.211 (95% CI 0.0688-0.645). The AUC was 0.965 (95% CI 0.945-0.978).

Removing one study [34] employing medical students and interns for smartphone ophthalmoscopy led to the greatest decrease in ρ to −0.639, indicating that the remaining studies fitted well within the SROC curve. However, removing the study utilizing indirect ophthalmoscopy [40] resulted in both a decrease in ρ and I^2^ to −0.464 and 93.8% (95% CI 88.5%-99.2%), respectively. Thus, our subgroup analysis for *severe NPDR* was inconclusive.

#### Proliferative Diabetic Retinopathy

A total of five studies (677 participants) presented data on detecting PDR [34,35,37,39,40]. DOR was 1225 (95% CI 117-12,800; I^2^_DOR_=78.0%; χ²_4_=18.2). Sensitivity and specificity ranged from 72% to 100% and 94% to 100%. The pooled sensitivity was 92.1% (95% CI 79.1%-97.4%); pooled specificity was 99.0% (95% CI 96.1%-99.8%). LR+ was 96.6 (95% CI 21.9-425); LR− was 0.0789 (95% CI 0.0273-0.228). The AUC was approximately 0.979.

Removing one study [34] decreased I^2^_DOR_ to 0.0%. This study employed a medical student and an intern to acquire smartphone ophthalmoscopy images, potentially resulting in heterogeneity. Removing the only study [35] using 7-field ETDRS fundus photography as a reference standard, or another study [40] utilizing indirect ophthalmoscopy, did not reduce I^2^_DOR_.

#### Diabetic Macular Edema

Although the diagnosis of DME generally requires stereoscopic retinal imaging, these studies used substitute markers, such as the presence of hard exudates or laser photocoagulation scars.

In all, four studies (627 participants) presented data on detecting DME [34,35,37,39]. I^2^_LRT_ was 87.9% (95% CI 75.5%-100%), χ²_3_=16.6, and ρ=−0.038. DOR was 49.8 (95% CI 13.7–180). Sensitivity and specificity ranged from 47% to 87% and 76% to 98%, respectively. The pooled sensitivity was 79.2% (95% CI 63.2%-89.4%); pooled specificity was 92.9% (95% CI 82.3%-97.4%). LR+ was 11.1 (95% CI 4.22-29.5); LR− was 0.224 (95% CI 0.119-0.422). The AUC was 0.925 (95% CI 0.898–0.945). Considering a pretest probability of 7.48% in diabetic patients [2], using the Fagan nomogram, the posttest probability for a positive and negative result was 47% and 2%, respectively.

#### Referral-Warranted Diabetic Retinopathy

 In all, four studies (313 participants) presented data on detecting RWDR [33,34,37,40]. I^2^_LRT_ was 94.3% (95% CI 89.6%-99.1%), χ²_3_=35.3, and ρ=−1.00. DOR was 75.8 (95% CI 13.9-414). Sensitivity and specificity ranged from 88% to 93% and 57% to 98%, respectively. The pooled sensitivity was 90.5% (95% CI 85.5%-93.8%); pooled specificity was 88.9% (95% CI 56.2%-98.0%). LR+ was 8.13 (95% CI 1.63-40.5); LR− was 0.107 (95% CI 0.0721-0.159). The AUC was 0.921 (95% CI 0.894-0.941).

#### Referral-Warranted Diabetic Retinopathy, Vision-Threatening Diabetic Retinopathy, and Sight-Threatening Diabetic Retinopathy

Overall, six studies (914 participants) presented data on detecting RWDR, VTDR, and STDR [33-35,37,38,40]. I^2^_LRT_ was 98.6% (95% CI 97.8%-99.4%), χ²_5_=139, and ρ=−1.00. DOR was 171 (95% CI 25.9-1142). Sensitivity and specificity ranged from 59% to 93% and 57% to 100%, respectively. The pooled sensitivity was 86.5% (95% CI 77.1%-92.4%); pooled specificity was 96.4% (95% CI 71.1%-99.7%). LR+ was 24.1 (95% CI 2.58-226); LR− was 0.140 (95% CI 0.0865-0.228). The AUC was 0.929 (95% CI 0.903-0.949). Owing to a good fit of the SROC curve, subgroup analysis was not performed.

One study excluded from the analysis found the agreement for detecting VTDR to be high, κ=0.76 (95% CI 0.68-0.85) [39].

#### Artificial Intelligence in Smartphone Ophthalmoscopy

In all, two studies (152 participants) presented data on detecting RWDR using AI to grade retinal images acquired via smartphone ophthalmoscopy compared with conventional slit-lamp biomicroscopy [32,33]. I^2^_LRT_ was 0.0%. DOR was 11.3 (95% CI 4.92-26.1). Specificity ranged from 46% to 57%. The pooled sensitivity was 91.2% (95% CI 84.3%-95.7%); pooled specificity was 50.0% (95% CI 38.3%-61.7%). LR+ was 1.80 (95% CI 1.42-2.28); LR− was 0.167 (95% CI 0.088-0.316). Owing to the limited number of included studies, the fixed effects model Moses-Littenberg SROC curve was employed for this analysis, and a 95% confidence region was not available.

Another study (301 participants) compared an AI’s grading of smartphone ophthalmoscopy images with the reference standard ophthalmologists’ grading of the same images [36]. It reported a high sensitivity of 95.8% (95% CI 92.9%-98.7%), 99.1% (95% CI 95.1%-99.9%), and 99.3% (95% CI 96.1%-99.9%), and a specificity of 80.2% (95% CI 72.6%-87.8%), 80.4% (95% CI 73.9%-85.9%), and 68.8% (95% CI 61.5%-76.2%) for any DR, STDR, and RWDR, respectively.

## Discussion

### Summary of Results

Overall, smartphone ophthalmoscopy performed well in detecting DR. Depending on the severity of DR, smartphone ophthalmoscopy had different accuracy. Progressing from mild NPDR to PDR, we observed an increasing trend in smartphone ophthalmoscopy’s sensitivity, specificity, and DOR. In addition, smartphone ophthalmoscopy had the best performance in detecting PDR, RWDR, VTDR, and STDR; these are important categories to detect as they can significantly affect vision. The lowest sensitivity was observed for detecting mild NPDR, mainly caused by one study enrolling only 7 participants with RWDR. The DOR was lowest for AI’s detection of RWDR. There was also a low percentage of ungradable images across most studies, implying that smartphone ophthalmoscopy is relatively reliable. Common causes of ungradable images included cataracts, poor pupil dilation, vitreous hemorrhages, or poor image focus.

Most studies performed smartphone direct ophthalmoscopy utilizing one of four different attachments. The included studies also assessed two methods of indirect ophthalmoscopy. Smartphone ophthalmoscopy in the included studies surpasses the UK NHS targets requiring DR retinal imaging equipment to have a minimum resolution of 6 megapixels or 30 pixels per retinal degree [34,37]. Two studies [35,36] assigned DR grades at a patient level instead of assessing each eye individually. In some cases, smartphone apps were used to digitally stitch the multiple images obtained per eye or enhance the image acquisition process by facilitating ergonomic focusing and capturing of images. Videography was used in two studies [37,38]. Different grading criteria and reference standards were applied across the included studies. High heterogeneity among studies was observed for most types of DR—especially for *moderate NPDR* and *PDR*—except for studies employing AI to detect RWDR. In other studies reporting on the use of non-AI smartphone ophthalmoscopy in *mild NPDR*, *RWDR only*, and *RWDR, VTDR, and STDR*, a significant proportion of heterogeneity can be attributed to the threshold effect.

The diagnostic accuracy of AI in grading smartphone ophthalmoscopy images was unexpectedly low. In two studies, the specificity and DOR of AI in detecting RWDR was lower than that of human graders (retinal specialists and ophthalmologists). Nevertheless, one of those studies employed both human and AI to grade identical smartphone-acquired images; the specificity of AI was higher than that of humans. In contrast, a 2015 study demonstrated that AI detects RWDR in smartphone ophthalmoscopy images with 100% sensitivity and 80% specificity (AUC 0.94) [41]. In addition, a recent review revealed that AI software achieved high sensitivity and specificity for detecting DR in datasets of fundus images acquired from other imaging modalities [42]. Finally, IDx-DR was the first commercially approved AI-based autonomous diagnostic system for DR detection. In a prospective study of 900 participants, this system attained high sensitivity and specificity of 87.2% (95% CI 81.8%-91.2%) and 90.7% (95% CI 88.3%-92.7%), respectively, in detecting more than mild DR [43].

Smartphone ophthalmoscopy is a safe means of acquiring retinal images [44]. One study [34] surveyed patients on their comfort levels while undergoing retinal imaging and revealed that all participants felt more comfortable with the light from Cellscope Retina than the light from slit lamps. Other studies [38,39] employing either an intrinsic smartphone light source or external light sources reported lower luminance than conventional fundus cameras.

### Comparison to Existing Studies

To our knowledge, this is the first meta-analysis evaluating the diagnostic accuracy of smartphone ophthalmoscopy for detecting DR in diabetic patients. A meta-analysis [45] evaluated the agreement between smartphone retinal imaging and retinal cameras encompassing multiple eye pathologies such as DR, glaucoma, and ocular hypertension. It reported excellent image quality in 84.7% of smartphone images, with good diagnostic accuracy; combined κ agreement was 77.8% (95% CI 70.34%-83.70%), AUC=0.86. However, the patient selection was not limited to diabetic individuals. A large study [46] involving 1460 participants (2920 eyes) had previously evaluated the diagnostic accuracy of smartphone ophthalmoscopy for optic disc imaging. Videography was performed with Peek Retina adaptor attached to an 8.0-megapixel Samsung SIII smartphone. This technique demonstrated excellent agreement (weighted κ=0.69) with a reference standard tabletop fundus camera in measuring vertical cup-disc ratio. Using smartphone ophthalmoscopy, 79.5% of eyes were gradable, compared with 86.4% for tabletop retinal imaging. Furthermore, there was no significant difference between image quality acquired by professional and inexperienced photographers. This study reported a lower percentage of gradable eyes compared with most studies included in our scoping review. This could be attributed to inherent differences in the process of DR grading (which requires examination of the retina in general) compared with measuring cup-disc ratio (which specifically examines the optic disc). Regardless of sample size, the agreement of smartphone ophthalmoscopy with a well-established reference standard remains high.

### Strengths

This scoping review aimed to provide a comprehensive analysis of the available literature in this field. Correspondingly, we had broad inclusion criteria encompassing different smartphone ophthalmoscopy techniques, reference standards, DR severity scales, and health care professionals. Smartphone retinal imaging is an emerging technology, and we wanted to capture as much of the available evidence as possible (Multimedia Appendix 5). The included studies were published relatively recently, from 2015 to 2018, heralding future breakthroughs in the diagnostic accuracy of smartphone retinal imaging as affordable and accessible means of DR detection.

Our study employed a comprehensive search strategy and examined studies from different countries involving different types of diabetic patients. At least two reviewers performed quality assessment and data extraction independently following Cochrane methodology. Based on the QUADAS-2 tool, most studies possessed minimal risk of bias and little applicability concerns. In particular, all included studies blinded or masked the graders.

### Limitations

Although the protocol for this scoping review was published in *BMJ Open*, this protocol was not registered. Three studies [33,34,39] utilized two graders, thereby creating two separate 2×2 tables; to avoid double-counting, we averaged the TP, FP, TN, and FN values, and rounded those average values to the nearest whole number for analysis. For one study [39], we assumed none of the four excluded eyes had DME. Owing to the small number of studies and limited information available, we were not able to conduct a meta-regression analysis or assess for publication bias.

The large 95% CIs for most SROC curves indicate imprecision. Although only studies involving diabetic patients were included, most studies were conducted in tertiary health care settings: eye or diabetes clinics. These settings can afford tabletop or portable fundus cameras. Instead, smartphone ophthalmoscopy is more relevant for screening in primary settings or resource-constrained countries. All studies required mydriasis, despite the availability of nonmydriatic smartphone ophthalmoscopy attachments [17].

### Implications for Future Research

Future studies on smartphone ophthalmoscopy could utilize more consistent reference standards, such as the gold standard 7-field ETDRS stereoscopic color photographs, and standardize the DR classification criteria. Such standardization minimizes bias and heterogeneity between studies. In addition, ultrawide-field retinal imaging may detect DR features outside the 7-field ETDRS field of view, which may be of clinical significance [47]. More studies could focus on (1) indirect ophthalmoscopy or ultrawide-field retinal imaging; (2) nonmydriatic techniques; (3) AI; and (4) primary health care settings where the comorbidities and prevalence of DR in this demographic differs.

### Conclusions

Smartphone ophthalmoscopy may have an important role in identifying DR in areas with limited access to expensive retinal imaging equipment and trained staff. Our findings show that smartphone ophthalmoscopy performs well in detecting DR. However, the included studies were scarce and heterogeneous and provided imprecise findings. Future studies should use more consistent reference standards and DR classification criteria, evaluate other available forms of smartphone ophthalmoscopy, and recruit participants from primary care settings.

## Appendix

Multimedia Appendix 1 Medical Literature Analysis and Retrieval System Online (MEDLINE), EMBASE, and Cochrane Library search strategy.

Multimedia Appendix 2 Data extraction sheet.

Multimedia Appendix 3 Additional details of included studies.

Multimedia Appendix 4 Details of quality assessment of included studies using Quality Assessment of Diagnostic Accuracy Studies-2.

Multimedia Appendix 5 Additional study details received from study investigators.

## Abbreviations

AI: artificial intelligence
AUC: area under curve
DM: diabetes mellitus
DME: diabetic macular edema
DOR: diagnostic odds ratio
DR: diabetic retinopathy
ETDRS: Early Treatment Diabetic Retinopathy Study
FN: false negatives
FP: false positives
LR−: negative likelihood ratio
LR+: positive likelihood ratio
LRT: likelihood ratio test
NHS: National Health Service
NPDR: nonproliferative diabetic retinopathy
PDR: proliferative diabetic retinopathy
QUADAS: Quality Assessment of Diagnostic Accuracy Studies
RWDR: referral-warranted diabetic retinopathy
SROC: summary receiver operating characteristic
STDR: sight-threatening diabetic retinopathy
TN: true negatives
TP: true positives
VTDR: vision-threatening diabetic retinopathy
