# Supplementary material for: Use of Smartphones to Detect Diabetic Retinopathy: Scoping Review and Meta-Analysis of Diagnostic Test Accuracy Studies
Source: J Med Internet Res. 2020 May 15;22(5):e16658. doi: 10.2196/16658 (PMC7316182; doi:10.2196/16658)
Supplement: Multimedia Appendix 1 [file jmir_v22i5e16658_app1.pdf]

Supplementary Data 1

eTable 1. MEDLINE (Ovid) search strategy

Concept	Search terms (combined by OR)
A. Target condition	1. exp RETINA/ or retin*.mp.<br>2. exp EYE DISEASES/ or exp EYE/ or eye*.mp.<br>3. exp BLINDNESS/ or blind*.mp.<br>4. exp Macular Edema/ or macular ?edema.mp.<br>5. exp Vision Disorders/ or (vis* adj3 (loss or impair* or defect* or disorder*)).mp.<br>6. maculopath*.mp.<br>7. exp OPHTHALMOLOGY/ or exp OPHTHALMOSCOPY/ or (fundoscop* or ophthalmo*).mp.<br>8. (fundus adj3 (imag* or photo* or video* or camera*)).mp.
B. Diagnostic test	1. exp smartphone/<br>2. exp CELL PHONE/<br>3. (smartphon* or cellphon* or phon* or mobile* or cell-phon* or smart-phon* or mobilephon* or mobile-phon*).mp.<br>4. exp Artificial Intelligence/<br>5. exp Machine Learning/<br>6. exp Pattern Recognition, Automated/<br>7. exp "Neural Networks (Computer)"/<br>8. artificial intelligen*.mp.<br>9. AI.mp.<br>10. Machine learning.mp.<br>11. Deep learning.mp.
C. Participant description	1. diabet*.mp.<br>2. exp Diabetes Mellitus/<br>3. exp HYPERGLYCEMIA/ or hyperglyc?emi*.mp.<br>4. exp Glucose Intolerance/ or glucose intoleran*.mp.<br>5. ((high or elevat*) adj3 (blood sugar or blood glucose)).mp.
**D. Overall search**	(A and B and C) or (exp diabetic retinopathy/ and B)
**Limits**	limit D to yr="2000 -Current"

EMBASE (Ovid) search strategy

Database: Embase <1974 to 2018 November 21>
Search Strategy:

----

1 exp diabetic retinopathy/ (36990)
2 exp retina/ or retin*.mp. (462847)
3 exp eye disease/ or exp eye/ or eye*.mp. (1145962)
4 exp blindness/ or blind*.mp. (450214)
5 exp retina macula edema/ or exp macular edema/ or exp diabetic macular edema/ or macular ?edema.mp. (16527)
6 exp visual impairment/ or exp visual disorder/ or (vis* adj3 (loss or impair* or defect* or disorder*)).mp. (246595)
7 exp retina maculopathy/ or maculopath*.mp. (37070)
8 exp ophthalmology/ or exp ophthalmoscopy/ or exp visual system examination/ or (fundoscop* or ophthalmo*).mp. (211344)
9 (fundus adj3 (imag* or photo* or video* or camera*)).mp. (10933)
10 exp smartphone/ or exp mobile phone/ or (smartphon* or cellphon* or phon* or mobile* or cell-phon* or smart-phon* or mobilephon* or mobile-phon*).mp. (199853)
11 exp artificial intelligence/ or exp artificial neural network/ or artificial intelligenc*.mp. or AI.mp. (78221)
12 exp diabetes mellitus/ (845630)
13 diabet*.mp. (1007491)
14 exp hyperglycemia/ or hyperglyc?emi*.mp. (113523)
15 exp glucose intolerance/ or glucose intoleran*.mp. (21781)
16 ((high or elevat*) adj3 (blood sugar or blood glucose)).mp. (6936)
17 or/2-9 (1726298)
18 or/10-11 (276945)
19 or/12-16 (1057627)
20 (1 and 18) or (17 and 18 and 19) (816)
21 limit 20 to yr="2000 -Current" (752)

****

Cochrane Library search strategy

ID	Search
#1	MeSH descriptor: [Diabetic Retinopathy] explode all trees
#2	retin*
#3	eye*
#4	blind*
#5	macular ?edema
#6	vis* NEAR/3 (loss OR impair* OR defect* OR disorder*)
#7	maculopath*
#8	fundoscop* OR ophthalmo*
#9	fundus NEAR/3 (imag* OR photo* OR video* OR camera*)
#10	MeSH descriptor: [Retina] explode all trees
#11	MeSH descriptor: [Eye Diseases] explode all trees
#12	MeSH descriptor: [Eye] explode all trees
#13	MeSH descriptor: [Blindness] explode all trees
#14	MeSH descriptor: [Macular Edema] explode all trees
#15	MeSH descriptor: [Vision Disorders] explode all trees
#16	MeSH descriptor: [Ophthalmology] explode all trees
#17	MeSH descriptor: [Ophthalmoscopy] explode all trees
#18	smartphon* OR cellphon* OR phon* or mobile* OR cell-phon* OR smart-phon* OR mobilephon* OR mobile-phon*
#19	MeSH descriptor: [Smartphone] explode all trees
#20	MeSH descriptor: [Cell Phone] explode all trees
#21	artificial intelligen* OR AI OR machine learning OR deep learning
#22	MeSH descriptor: [Artificial Intelligence] explode all trees
#23	MeSH descriptor: [Machine Learning] explode all trees
#24	MeSH descriptor: [Neural Networks (Computer)] explode all trees
#25	MeSH descriptor: [Pattern Recognition, Automated] explode all trees
#26	diabet* OR hyperglyc?emi* OR glucose intoleran* OR ((high or elevat*) NEXT/3 (blood sugar or blood glucose))
#27	[mh "Diabetes Mellitus"] OR [mh Hyperglycemia] OR [mh "Glucose Intolerance"]
#28	{OR #2-#17}
#29	{OR #18-#25}
#30	{OR #26-#27}
#31	(#1 AND #29) OR (#28 AND #29 AND #30)
