# Supplementary material for: Use of Smartphones to Detect Diabetic Retinopathy: Scoping Review and Meta-Analysis of Diagnostic Test Accuracy Studies
Source: J Med Internet Res. 2020 May 15;22(5):e16658. doi: 10.2196/16658 (PMC7316182; doi:10.2196/16658)
Supplement: Multimedia Appendix 2 [file jmir_v22i5e16658_app2.pdf]

Supplementary Data 2

Data Extraction Sheet

Study Identification	Journal published<br>Publication type<br>Country of study<br>Setting of study
Study Design	Study methodology (consecutive or random; retrospective or prospective)<br>Start date<br>End date<br>DR severity scale used<br>Definition of RWDR, VTDR and/or STDR
Participants	Total number of participants included in study<br>Total number of participants excluded from study<br>Number of withdrawals<br>Sample size (patients)<br>Sample size (eyes)<br>Inclusion criteria<br>Exclusion criteria<br>Mean age and age range<br>Gender<br>Type of diabetes<br>Mean duration of diabetes<br>Spectrum of presenting symptoms and comorbidities<br>Current treatment
Index Test	Imaging technique<br>Attachment used (Y/N)<br>Type of attachment used<br>Weight of attachment<br>Type of smartphone used<br>Cost excluding smartphone<br>Fixation target<br>Light source<br>Mydriasis (Y/N)<br>Number of fields<br>Field of view (in degrees)<br>Fields/Regions of retina imaged<br>Stereoscopic (Y/N)<br>Colour (Y/N)<br>Image resolution<br>Healthcare professional acquiring retinal images<br>Healthcare professional grading images<br>Time taken to acquire images<br>Time taken to stitch images (if applicable)<br>Stitching software used (if applicable)<br>Other software used<br>Was AI used for grading retinal images? (Y/N)<br>AI software used for grading (if applicable)

Reference Standard	Type of reference standard<br>Healthcare professional performing reference standard<br>Time between index test and reference standard<br>Blinding (Y/N)
2×2 Table	TP<br>TN<br>FP<br>FN
Sensitivity (95% CI)	No DR<br>Mild NPDR<br>Moderate NPDR<br>Severe NPDR<br>Very severe NPDR<br>Early PDR<br>High-risk PDR<br>Severe PDR<br>Macular oedema<br>Others
Specificity (95% CI)	No DR<br>Mild NPDR<br>Moderate NPDR<br>Severe NPDR<br>Very severe NPDR<br>Early PDR<br>High-risk PDR<br>Severe PDR<br>Macular oedema<br>Others
PPV and NPV	Positive Predictive Value (DR)<br>Negative Predictive Value (DR)<br>Positive Predictive Value (DME)<br>Negative Predictive Value (DME)
Image quality	Categories (e.g. Excellent, moderate…)<br>Number of images in each category<br>Number of ungradable images
Graders	Number of graders<br>Number of eyes/patients assessed by each grader<br>Agreement between graders

Quality assessment of included studies using QUADAS-2

Patient Selection	Description
	Was a consecutive or random sample of patients enrolled?<br>Was a case-control design avoided?<br>Did the study avoid inapprorpiate exclusions?
	Risk of bias (high/ low/ unclear)
	Applicability Concerns
Index Test	Description
	Were the index test results interpreted without knowledge of the results of the reference standard?<br>If a threshold was used, was it prespecified?
	Risk of bias (high/ low/ unclear)
	Applicability Concerns
Reference Standard	Description
	Is the reference standard likely to correctly classify the target condition?<br>Were the reference standard results interpreted without knowledge of the results of the index test?
	Risk of bias (high/ low/ unclear)
	Applicability Concerns
Flow and Timing	Description
	Was there an appropriate interval between index tests and reference standards?<br>Did all patients receive a reference standard?<br>Were all patients included in the analysis?
	Risk of bias (high/ low/ unclear)

Source: Whiting et al, 2011. QUADAS-2: A Revised Tool for the Quality Assessment of Diagnostic Accuracy Studies
