# Supplementary material for: Use of Smartphones to Detect Diabetic Retinopathy: Scoping Review and Meta-Analysis of Diagnostic Test Accuracy Studies
Source: J Med Internet Res. 2020 May 15;22(5):e16658. doi: 10.2196/16658 (PMC7316182; doi:10.2196/16658)
Supplement: Multimedia Appendix 3 [file jmir_v22i5e16658_app3.pdf]

Supplementary Data 3

eTable 2. Participant inclusion and exclusion criteria

Study ID	Inclusion criteria	Exclusion criteria
Bhat 2016	Not available	Not available
Kim 2017	Not available	Not available
Kim 2018	1. Age ≥18 years.<br>2. No obvious eye media opacity (e.g. vitreous hemorrhage, advanced cataract).	-
Rajalakshmi 2015	1. Age >18 years.<br>2. Type 2 DM.<br>3. No contraindication to mydriasis (i.e. allergy to tropicamide).<br>4. Willing to undergo fundus imaging with two cameras.	-
Rajalakshmi 2018	1. Age ≥18 years.<br>2. Type 2 DM.	-
Russo 2015	1. Type 1 or 2 DM.	-
Ryan 2015	1. Age 18–65 years.<br>2. Type 2 DM.<br>3. Willing to undergo fundus imaging with three cameras.	1. Contraindication to mydriasis.<br>2. Overt media opacity.<br>3. Gestational diabetes.
Sengupta 2018	1. Age >21 years.	1. Patients with significant corneal or lenticular pathology precluding fundus examination.<br>2. Patients who had undergone previous vitreoretinal incisional or laser surgery.<br>3. Vitreous hemorrhage and cataract were NOT exclusion criteria if retina specialists could confidently visualize optic disc and detect retinal features (e.g. neovascularization).
Toy 2016	1. Adult patients.<br>2. Diabetic patients referred to clinic for DR screening.	-

eTable 3. Recruitment duration

Study ID	Start date	End date	Recruitment duration
Bhat 2016			
Kim 2017			
Kim 2018			
Rajalakshmi 2015	December 2014	February 2015	3 months
Rajalakshmi 2018			
Russo 2015			
Ryan 2015			5 months
Sengupta 2018	April 2015	January 2016	10 months
Toy 2016	February 2014	September 2014	8 months

eTable 4. Study setting

Study ID	Setting
Bhat 2016	-
Kim 2017	University of Michigan Kellogg Eye Center Retina Clinic.
Kim 2018	1. University of Michigan Kellogg Eye Center Retina Clinic.<br>2. Ophthalmology consultation service at the University of Michigan Hospital.<br>*Participants were recruited while undergoing routine examination.*
Rajalakshmi 2015	Eye department of tertiary care diabetes hospital network, Chennai.<br>*Participants were referred or presented for screening.*
Rajalakshmi 2018	
Russo 2015	Ophthalmic Diabetic Center of “Spedali Civili di Brescia” (outpatient clinic).<br>*Participants were recruited after routine examination during their first visit.*
Ryan 2015	Eye department of Dr Mohan’s Diabetes Specialties Centre, Chennai.
Sengupta 2018	Aravind Eye Hospitals retina clinic.
Toy 2016	Healthcare safety-net ophthalmology clinic, Santa Clara Valley Medical Center (SCVMC).<br>*Patients were referred by primary physicians or SCVMC diabetes clinic for DR screening.*

eTable 5. Further characteristics of included studies

Study ID	Power calculation	Unit of analysis
Bhat 2016	-	Patient-level, i.e. worse eye determines final diagnosis of each patient.
Kim 2017	-	Eye-level: Each eye assessed independently.<br>Patient-level: based on the worse eye.
Kim 2018	-	Each eye assessed independently.
Rajalakshmi 2015	Sample size calculated using a pilot study conducted on 50 patients.	Patient-level, i.e. worse eye determines final diagnosis of each patient.
Rajalakshmi 2018		
Russo 2015	-	Each eye assessed independently.
Ryan 2015	Sample size determined via duration of diabetes:<br>A. <18 months: 100 patients.<br>B. 18 months–15 years: 100 patients.<br>C. >15 years: 100 patients.	Two methods employed to calculate sensitivity and specificity:<br>1. Each eye assessed independently (raw).<br>2. Estimates accounting for inter-eye correlation (correlated).
Sengupta 2018	Sample size ≥200 allowed:<br>A. Measured discordance rate between Remidio FOP and Topcon to vary from true discordance rate by ≤7% (assuming true discordance rate of 10%–50%).<br>B. Sufficient representation of all DR grades.	Each eye assessed independently.
Toy 2016	Authors’ previous “Nonmydriatic fundus photography program” determined a 17% prevalence of DR. Thus, this sample size of 100 had 80% power to detect ≈10% disparity (<5.3% or >26% prevalence) in DR detection between screening methods.	Each eye assessed independently.

eTable 6. Study funding sources or conflicting interests

Study ID	Funding sources/ conflicting interests
Bhat 2016	Not available
Kim 2017	Not available
Kim 2018	**Grant information:**<br>• Michigan Translational Research and Commercialization program with support from MEDC and the UM Medical School.<br>• NEI grant no. K23 EY023596.<br>• QB3 Bridging the Gap Awards from the Rogers Family Foundation.<br>• Bakar Fellows Award.<br>• Corresponding author is a Chan-Zuckerberg Biohub investigator.
Rajalakshmi 2015	NIL
Rajalakshmi 2018	NIL
Russo 2015	NIL
Ryan 2015	NIL
Sengupta 2018	NIL
Toy 2016	**D. J. Myung:** Consultant for and equity holder of DigiSight Technologies, Inc; Patent holder for EyeGo adapter; grant support from Spectrum/BioDesign, Stanford Society of Physician Scholars.<br>**L. He:** Patent holder for EyeGo adapter.<br>**R. T. Chang:** Patent holder for EyeGo adapter; grant support from Spectrum/BioDesign, Stanford Society of Physician Scholars.<br>**M. S. Blumenkranz:** Board member and equity holder of DigiSight Technologies, Inc.<br>**A. Polkinhorne:** Consultant for and equity holder of DigiSight Technologies, Inc.<br>**D. J. Myung:** Employed by and equity holder of DigiSight Technologies, Inc.<br>**D. Foster:** Board member of, employed by, and equity holder of DigiSight Technologies, Inc.
