# Supplementary material for: Use of Smartphones to Detect Diabetic Retinopathy: Scoping Review and Meta-Analysis of Diagnostic Test Accuracy Studies
Source: J Med Internet Res. 2020 May 15;22(5):e16658. doi: 10.2196/16658 (PMC7316182; doi:10.2196/16658)
Supplement: Multimedia Appendix 4 [file jmir_v22i5e16658_app4.pdf]

Supplementary Data 4

eTable 7: Details of quality assessment of included studies using QUADAS-2

Study ID		Bhat 2016	Kim 2017	Kim 2018	Rajalakshmi 2015	Rajalakshmi 2018	Russo 2015	Ryan 2015	Sengupta 2018	Toy 2018
**Patient Selection**	Was a consecutive or random sample of patients enrolled?	Y	?	?	Y	?	Y	?	Y	Y
	Was a case-control design avoided?	Y	Y	Y	Y	Y	Y	Y	Y	Y
	Did the study avoid inappropriate exclusions?	?	?	Y	Y	Y	Y	Y	Y	Y
	Risk of bias	Unclear	Unclear	Unclear	Low	Low	Low	Unclear	Low	Low
	Applicability Concerns	Unclear	Good	Good	Good	Good	Good	Good	Good	Good
**Index Test**	Were the index test results interpreted without knowledge of the results of the reference standard?	Y	Y	Y	Y	Y	Y	Y	Y	Y
	If a threshold was used, was it prespecified?	NA	NA	NA	NA	NA	NA	NA	NA	NA
	Risk of bias	Low	Low	Low	Low	Low	Low	Low	Low	Low
	Applicability Concerns	Good	Good	Good	Good	Good	Good	Good	Good	Good
**Reference Standard**	Is the reference standard likely to correctly classify the target condition?	Y	Y	Y	Y	?	Y	Y	Y	Y
	Were the reference standard results interpreted without knowledge of the results of the index test?	Y	Y	Y	Y	Y	Y	Y	Y	Y
	Risk of bias	Low	Low	Low	Low	Unclear	Low	Low	Low	Low
	Applicability Concerns	Good	Good	Good	Good	Poor	Good	Good	Good	Good
**Flow and Timing**	Was there an appropriate interval between index tests and reference standards?	?	?	Y	Y	Y	Y	Y	Y	Y
	Did all patients receive a reference standard?	Y	Y	Y	Y	Y	Y	Y	Y	Y
	Were all patients included in the analysis?	?	N	N	Y	N	Y	Y	N	Y
	Risk of bias	Unclear	Unclear	Unclear	Low	Low	Low	Low	Low	Low
