# Supplementary material for: Use of Smartphones to Detect Diabetic Retinopathy: Scoping Review and Meta-Analysis of Diagnostic Test Accuracy Studies
Source: J Med Internet Res. 2020 May 15;22(5):e16658. doi: 10.2196/16658 (PMC7316182; doi:10.2196/16658)
Supplement: Multimedia Appendix 5 [file jmir_v22i5e16658_app5.pdf]

**Supplementary Data 5**

**eTable 8.** Additional study details received from study investigators

<table>
<tr><th>Study ID</th><th>Additional details received</th></tr>
<tr><td>Bhat 2016</td><td>Please see the following publications for more information on the device (FOV, camera resolution, iPhone used etc)<br>https://www.ncbi.nlm.nih.gov/pmc/articles/PMC6166894/<br>https://patents.google.com/patent/WO2015054672A1/en<br>https://www2.eecs.berkeley.edu/Pubs/TechRpts/2014/EECS-2014-91.html<br><br>Consecutive patients with Type I or II diabetes who consented were included in the study. Retinal images were captured by healthcare professionals (medical assistants, interns etc) and analyzed by the EyeArt AI eye screening system v1.2. Duration between acquiring images and doing slit lamp is unavailable.<br><br>Of the 80 patients, 32 patients had signs of referable DR (moderate NPDR or higher on ICDR scale and/or presence of CSME) as per the ground truth.</td></tr>
<tr><td>Kim 2017</td><td>Manuscript for this abstract was at that point in publication.</td></tr>
<tr><td>Kim 2018</td><td>No reply from corresponding author after two weeks.</td></tr>
<tr><td>Rajalakshmi, 2015</td><td>Data for 2×2 tables – true positives, false positives, true negatives, false negatives.<br><br>NB: This study employed a different methodology to calculate the sensitivity and specificity of smartphone ophthalmoscopy compared to other studies. To ensure consistency, we used data from this table instead:<br>Table 1. Diabetic retinopathy (DR) severity based on Fundus on phone (FOP) and Zeiss retinal photography
<table>
<tr><th>FOP CAMERA</th><th></th><th></th><th>ZEISS CAMERA</th><th></th><th></th><th></th></tr>
<tr><th></th><th>No DR n (%)</th><th>Mild NPDR n (%)</th><th>Moderate NPDR n (%)</th><th>Severe NPDR n (%)</th><th>PDR n (%)</th><th>Total</th></tr>
<tr><td>No DR n (%)</td><td>121 (40.2)</td><td>9 (3.0)</td><td>4 (1.3)</td><td>0</td><td>0</td><td>134 (44.5)</td></tr>
<tr><td>Mild NPDR n (%)</td><td>1 (0.3)</td><td>29 (9.7)</td><td>6 (2.0)</td><td>2(0.7)</td><td>1 (0.3)</td><td>39 (13.0)</td></tr>
<tr><td>Moderate NPDR n (%)</td><td>1 (0.3)</td><td>8 (2.7)</td><td>43 (14.3)</td><td>5 (1.7)</td><td>1 (0.3)</td><td>58 (19.3)</td></tr>
<tr><td>Severe NPDR n (%)</td><td>0</td><td>1 (0.3)</td><td>7 (2.3)</td><td>14 (4.7)</td><td>2 (0.7)</td><td>24 (8)</td></tr>
<tr><td>PDR n (%)</td><td>0</td><td>0</td><td>3 (1.0)</td><td>2 (0.7)</td><td>41 (13.6)</td><td>46 (15.3)</td></tr>
<tr><td>TOTAL</td><td>123 (40.9)</td><td>47(15.6)</td><td>63 (20.9)</td><td>22(7.3)</td><td>46 (15.3)</td><td>301 (100)</td></tr>
</table>
Table retrieved from: Rajalakshmi R, Arulmalar S, Usha M, Prathiba V, Kareemuddin KS, Anjana RM, et al. Validation of Smartphone Based Retinal Photography for Diabetic Retinopathy Screening. PLoS One. 2015;10(9):e0138285. PMID: 26401839</td></tr>
<tr><td>Ryan, 2015</td><td>No reply from corresponding author after two weeks.</td></tr>
<tr><td>Sengupta, 2018</td><td>Reference standard:<br>Patients underwent a comprehensive dilated retinal examination which included indirect ophthalmoscopy and evaluation of the retina using a +90D lens on the slit lamp.<br><br>Sensitivity and specificity values for detection of Macula Edema (M1):<br>Since very few eyes had macular edema (DME) alone and not R2 or R3 disease, this was not evaluated separately. Irrespective of the DR status, for both graders, and for both imaging modalities, the sensitivity of detecting DME ranged between 82.5–91.5% and specificity was 78–80%.<br>• Sensitivity of detecting DME = 86.5% (95% CI = 82.5–91.6%)<br>• Specificity of detecting DME = 75.6% (95% CI = 72–80%)<br><br>The numbers are slightly lower than those for DR status because diagnosis of DME requires depth perception which can only be achieved with stereo-photographs. Other clues such as presence of hard exudates and microaneurysms near the fovea are used by graders to comment on presence of DME, while clinical examination allows depth perception.<br><br>Specificity of Remidio Fundus on Phone (FOP) in detecting R1, R2 and R3 diseases:<br>• R1 (83.4%, 95% CI = 78–87%)</td></tr>
</table>

| | |
|---|---|
| | • R2 (89.7%, 95% CI = 83–94%)<br>• R3 (98.7%, 95% CI = 95–100%)<br><br>**As 4 eyes were excluded from the Remidio FOP analysis, what were the gold standard diabetic retinopathy diagnoses for those 4 eyes?**<br>Answer: 2 out of 4 were R1 and another 2 were R2 disease.<br><br>**2×2 table for vision-threatening diabetic retinopathy (VTDR) and diabetic macular edema (DME):**<br>The actual data numbers (exact numbers of VTDR and DME) cannot be shared with any external sources as it is prohibited by the Ethics committee. |
| Toy 2016 | **In the Results section, smartphone-acquired fundus images were compared to the "clinical grade as a reference (standard)". What specific tests were performed to determine the clinical grades?**<br><br>Clinical grade was determined by two masked graders based on the International Clinical Classification for Diabetic Retinopathy (ICDR) disease severity scale.<br><br>**Was there any unpublished data related to sensitivity and specificity values of smartphone-acquired fundus photographs for any of the following conditions:**<br>• **Any form of diabetic retinopathy?**<br>• **Any specific grade/type of diabetic retinopathy (mild/moderate/severe non-proliferative or proliferative)?**<br><br>Using clinical grade as the reference to detect referral-warranted retinopathy, photograph grade was found to be 91% sensitive and 99% specific, with a 95% positive predictive value and a 98% negative predictive value. The requested sensitivity and specificity values for the various grades can be calculated from our Table 3 and Table 4. Please note that the number of patients in some of the categories were small, so caution is advised in drawing definitive conclusions from the subgroups. |
